# Structure model of γ-Al_2_O_3_ based on planar defects

**DOI:** 10.1107/S2052252518015786

**Published:** 2019-01-01

**Authors:** Martin Rudolph, Mykhaylo Motylenko, David Rafaja

**Affiliations:** a Institute of Materials Science, TU Bergakademie Freiberg, Gustav-Zeuner-Straße 5, D–09599 Freiberg, Germany

**Keywords:** γ-alumina, microstructure defects, antiphase boundaries, rotational boundaries, selected-area electron diffraction, powder X-ray diffraction, Debye equation, anisotropic broadening

## Abstract

The defect structure of γ-Al_2_O_3_ derived from boehmite was investigated using a combination of selected-area electron diffraction (SAED) and powder X-ray diffraction (XRD). Both methods confirmed a strong dependence of the diffraction line broadening on the diffraction indices known from literature. The analysis of the SAED patterns revealed that the dominant structure defects in the spinel-type γ-Al_2_O_3_ are antiphase boundaries located on the lattice planes , which produce the sublattice shifts . Quantitative information about the defect structure of γ-Al_2_O_3_ was obtained from the powder XRD patterns. This includes mainly the size of γ-Al_2_O_3_ crystallites and the density of planar defects. The correlation between the density of the planar defects and the presence of structural vacancies, which maintain the stoichiometry of the spinel-type γ-Al_2_O_3_, is discussed. A computer routine running on a fast graphical processing unit was written for simulation of the XRD patterns. This routine calculates the atomic positions for a given kind and density of planar defect, and simulates the diffracted intensities with the aid of the Debye scattering equation.

## Abstract 2

The defect structure of γ-Al_2_O_3_ produced from boehmite is described with the aid of antiphase and rotational boundaries. The type of structure defects is deduced from the selected-area electron-diffraction patterns. The defect density is quantified from the anisotropic broadening of diffraction lines in powder X-ray diffraction patterns using a computer routine based on the Debye scattering equation.

## 1. Introduction   

γ-Alumina (γ-Al_2_O_3_) is one of the intermediate aluminium oxides that accompany the temperature-induced transition of boehmite [γ-AlO(OH)] towards the thermodynamically stable corundum (α-Al_2_O_3_), see Euzen *et al.* (2002 ▸):
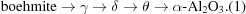
Because of its large surface area and high catalytic activity, metastable γ-Al_2_O_3_ is often used as a functional constituent of catalytic converters or surface coatings. However, as γ-Al_2_O_3_ is only stable below 700^o^C (Paglia *et al.*, 2004 ▸; Rudolph *et al.*, 2017 ▸), it must be stabilized for high-temperature applications.

All intermediate alumina phases possess a slightly distorted cubic close-packed (c.c.p.) sublattice of oxygen anions. This sublattice is already present in the octahedral double layers of boehmite (Christensen *et al.*, 1982 ▸; Wilson, 1979 ▸). It survives the topotactic phase transition 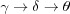 and is rearranged only by the formation of α-Al_2_O_3_ at approximately 1200^o^C (Euzen *et al.*, 2002 ▸). Aluminium cations occupy octahedral and tetrahedral sites in the crystal structures of most intermediate alumina phases. However, in contrast to the fully occupied oxygen sublattice, the aluminium sublattice contains structural vacancies, which balance the [Al]/[O] ratio. In consecutive intermediate alumina phases, these vacancies show different degrees of ordering that increases in general from γ-Al_2_O_3_ to θ-Al_2_O_3_.

The different distribution of vacancies on the cation sites is one of the reasons why different structural descriptions of γ-Al_2_O_3_ can be found in the literature for differently prepared samples, for samples with different crystallite size *etc.* Thus, there is a need for a generalized structure model of γ-Al_2_O_3_, which would take into account the presence of structural vacancies with partially correlated positions and which would allow for quantitative description of these structure defects. Such a structure model is a first step towards understanding the successive phase transformation in metastable alumina phases and a prerequisite for targeted manipulation of their thermal stability.

Zhou & Snyder (1991 ▸) described the crystal structure of γ-Al_2_O_3_ as a defective spinel structure. The term ‘defective’ stands for a small tetragonal distortion of the cubic lattice, for small static displacements of atoms from their ideal positions, for the presence of vacancies in the cation sublattice and for the displacement of some cations into the non-spinel sites. In an ideal spinel structure with the structural formula 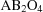 and with the space group 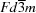, the O^2−^ anions occupy the Wyckoff positions 32*e* within the c.c.p. sublattice, the A^2+^ cations occupy the tetrahedral sites 8*a* and the B^3+^ cations occupy the octahedral sites 16*d* (Sickafus *et al.*, 2004 ▸). From the cation valency and from the cation-to-anion ratio (2/3) in Al_2_O_3_, it becomes evident that the trivalent Al^3+^ cations have to be located on both, tetrahedral and octahedral, sites. Moreover, a non-integer number of cation sites have to remain vacant to obey the stoichiometry of Al_2_O_3_.

In the structure models of γ-Al_2_O_3_ based on the analysis of integral intensities obtained from the X-ray or neutron powder diffraction patterns, the structural vacancies are typically randomly distributed over the standard tetrahedral and octahedral spinel sites (8*a *and 16*d*). In order to be able to reproduce the measured integral intensities more accurately, the sites 8*b*, 16*c* and 48*f*, which are empty in ideal spinel structures, were assumed to be partially occupied by Al^3+^ cations (Verwey, 1935 ▸; Ushakov & Moroz, 1984 ▸; Zhou & Snyder, 1991 ▸; Paglia *et al.*, 2003 ▸; Smrčok *et al.*, 2006 ▸). As a random distribution of vacancies at the cation sites can lead to an energetically unfavorable clustering of Al^3+^ cations (Cowley, 1953 ▸), specific ordering of vacancies is usually assumed when the local structure is investigated, *e.g.* in *ab initio* calculations (Digne *et al.*, 2004 ▸; Menéndez-Proupin & Gutiérrez, 2005 ▸).

However, none of the above crystal structure models can explain the observed dependence of the X-ray diffraction (XRD) line broadening on the diffraction indices, which was already reported by Zhou & Snyder (1991 ▸). Alternative microstructure models of γ-Al_2_O_3_ are based on the assumption that the anisotropy of the line broadening observed is caused by planar defects, which are known to broaden the diffraction lines differently even for equivalent *hkl* (Warren, 1990 ▸; Guinier, 1994 ▸). Typical examples of the planar defects reported in conjunction with the microstructure models of γ-Al_2_O_3_ are antiphase boundaries (Dauger & Fargeot, 1983 ▸) and stacking faults (Cowley, 1953 ▸; Fadeeva *et al.*, 1977 ▸; Paglia *et al.*, 2006 ▸; Tsybulya & Kryukova, 2008 ▸). In these models, a coalescence of vacancies at the planar defects was assumed in order to avoid unwanted occupation of neighboring cation lattice sites (Kryukova *et al.*, 2000 ▸).

Based on these considerations, Tsybulya & Kryukova (2008 ▸) and Pakharukova *et al.* (2017 ▸) developed a three-dimensional model of the real structure of γ-Al_2_O_3_, which consists of small unperturbed nano-sized domains of γ-alumina with spinel-like crystal structure. The nano-domains are terminated by the lattice planes {001}, {011} and {111} and possess polyhedral shapes. Neighboring nano-domains are mutually shifted along the gliding planes {001}, {011} and {111}. The shift vectors are summarized in Table 1 ▸.

The defects from Table 1 ▸ act as stacking faults for the cation sublattice, but they keep the anion sublattice intact. Consequently, they do not cause any broadening of the diffraction lines, which stem predominantly from the scattering of X-rays on the oxygen sublattice, whereas the diffraction lines produced by the scattering on the aluminium sublattice are strongly broadened. The capability of this model to explain the observed anisotropy of the XRD line broadening was recently illustrated by Pakharukova *et al.* (2017 ▸) for γ-Al_2_O_3_ derived from a boehmite-based aerogel precursor.

In the present study, we extend the microstructure models invented by Tsybulya & Kryukova (2008 ▸) and Pakharukova *et al.* (2017 ▸) by considering not only fractional lattice translations but also selected lattice rotations, and discuss the influence of resulting microstructure defects on selected-area electron diffraction (SAED) and XRD patterns. In all cases, only the planar defects are considered, which do not affect the atomic ordering within the oxygen sublattice.

The SAED patterns were simulated using *JEMS* (Stadelmann, 2012 ▸) and *DIFFaX* (Treacy *et al.*, 1991 ▸). For simulation of the XRD patterns, a fast algorithm based on the Debye equation (Debye, 1915 ▸) was written for a graphical processing unit. The simulated diffraction patterns were compared with the diffraction patterns measured on γ-Al_2_O_3_ that was obtained by annealing of highly crystalline boehmite. As the γ-alumina derived from boehmite exhibits a small tetragonal distortion, the defect crystal structure is described in space group 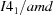 instead of 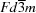 (Paglia *et al.*, 2003 ▸). For the orientation relationship between cubic and tetragonal γ-alumina, 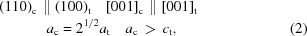
the equivalent lattice planes and Wyckoff positions are summarized in Table 2 ▸.

## 2. Experimental   

**Figure 1:**
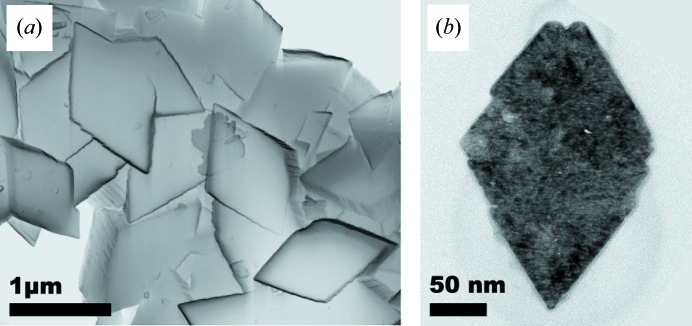
(*a*) Scanning electron micrograph of large boehmite particles in initial state and (*b*) transmission electron micrograph of a small particle in the annealed state.

The γ-Al_2_O_3_ under study was prepared by heating highly crystalline boehmite powder (Actilox^®^ B20, Nabaltec) in air for 20 h at 600^o^C. The heating rate was 2°C min^−1^. In the original state, the boehmite particles had the shape of thin, almost rhombic platelets (Fig. 1 ▸). Their size varies between a few hundred nanometres and several micrometres (Rudolph *et al.*, 2017 ▸). After annealing, this particle shape was preserved, but the corresponding crystallographic directions changed according to the orientation relationship 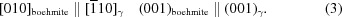
The SAED patterns were recorded using a JEM-2200 FS field-emission transmission electron microscope (TEM) that was operated at the acceleration voltage of 200 kV. The TEM was equipped with a high-resolution objective lens (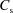 = 0.5 mm) and a corrector of the spherical aberration that was located in the primary beam. The XRD experiments were performed on a Bragg–Brentano diffractometer (URD6 from Seifert/FPM) using Cu *K*α radiation (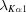 = 1.5406 Å, 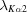 = 1.5444 Å) and a scintillation detector. The XRD patterns were collected in a 2θ range between 15 and 70° with a 0.02° step size and a counting time of 12 s per step. For calculation of the XRD patterns the same wavelengths and 2θ range were utilized.

## 3. Results   

### 3.1. Models of planar defects in γ-Al_2_O_3_ obtained from analysis of the SAED pattern   

**Figure 2:**
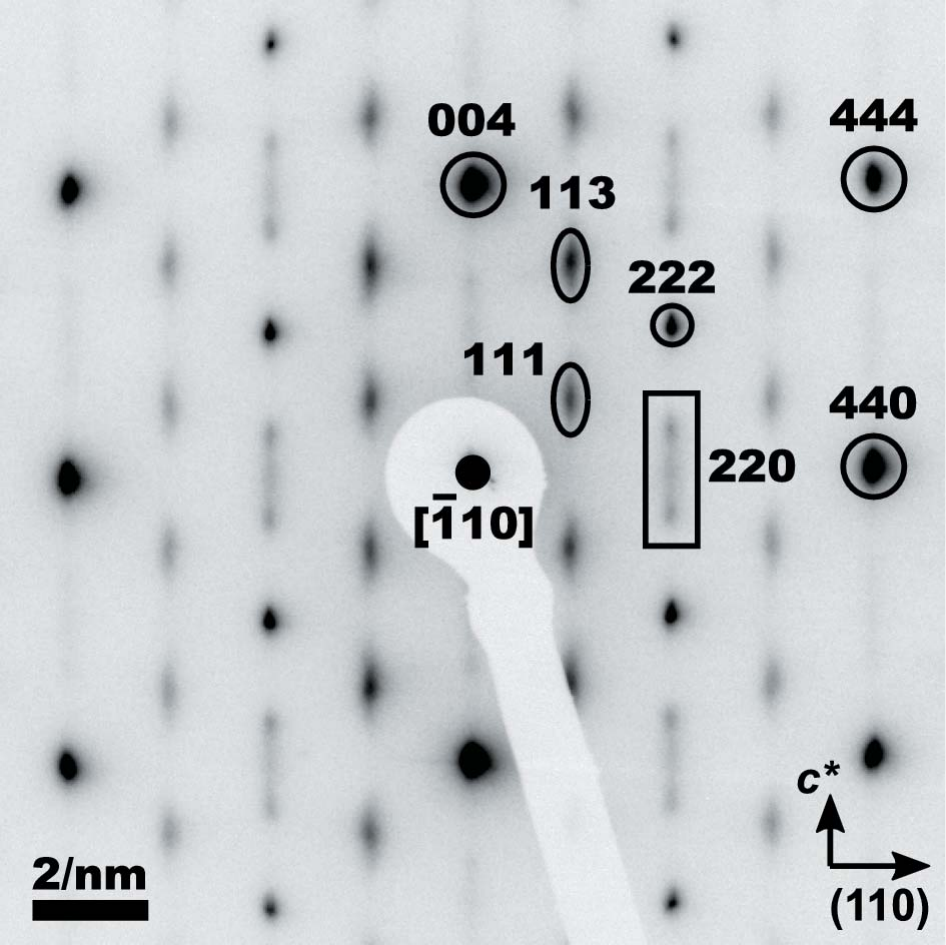
SAED pattern of γ-Al_2_O_3_. Diffraction indices are given for the cubic structure. The corresponding tetragonal diffraction indices are shown in Table 2 ▸.

SAED pattern of γ-Al_2_O_3_ derived from boehmite (Fig. 2 ▸) consists of narrow diffraction spots and streaks, which are elongated in the 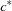 direction. This kind of line broadening indicates the presence of planar defects on the lattice planes 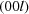, which was already described by Cowley (1953 ▸). The observed dependence of the line broadening on the diffraction indices confirms different degrees of disorder in individual sublattices. As it can be seen from equation (7), the strongly elongated diffraction spots (111, 113, 220, 224, 331, 333 *etc.*) stem from the disordered cation sublattice, while the narrow diffraction spots (004, 222, 440, 444 *etc.*) are dominated by the scattering on a fully occupied and well ordered c.c.p. anion sublattice (Lippens & de Boer, 1964 ▸; Paglia *et al.*, 2004 ▸; Tsybulya & Kryukova, 2008 ▸).

The difference in shape of the elongated diffraction spots with even (220, 224 *etc.*) and odd (111, 113, 331, 333 *etc.*) indices stems from different arrangements of Al^3+^ cations on regular (8*a* and 16*d*) and irregular (8*b*, 16*c* and 48*f*) Wyckoff sites. It follows from Table 4 and equation (7) that the structure factors of the reflections with odd diffraction indices depend on the occupancy of the regular Wyckoff positions 8*a*, 16*d* and the irregular Wyckoff positions 8*b* and 16*c*, while the structure factors of the reflections with even diffraction indices depend on the occupancy of the regular Wyckoff position 8*a* and on the occupancy of the irregular positions 8*b* and 48*f*.

The observed dependence of the line broadening on diffraction indices (Fig. 2 ▸) confirms that in γ-Al_2_O_3_ derived from highly crystalline boehmite, the anion sublattice remains intact, while the cation sublattice is disordered in such a way that the tetrahedral sites 8*a*, 8*b* and 48*f* with 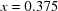 are more disordered than the octahedral positions 16*d* and 16*c*, as already proposed by Zhou & Snyder (1991 ▸) and Paglia *et al.* (2004 ▸).

**Figure 3:**
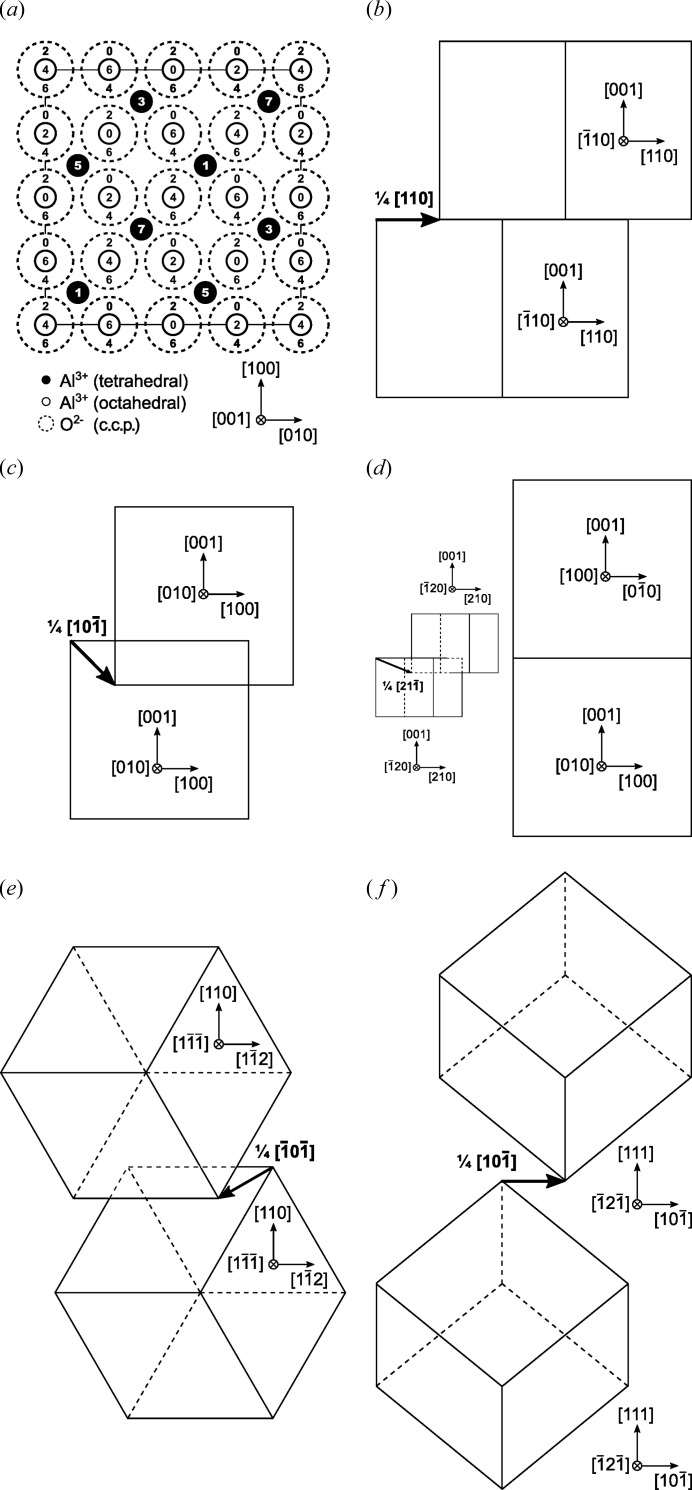
(*a*) Projected unit cell of an idealized (cubic) γ-Al_2_O_3_ spinel structure. The numbers located within the circles representing the atomic positions give the *z* coordinates in multiples of 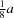. Further panels give an overview of considered planar defects: (*b*) conservative APB of the type 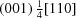, (*c*) non-conservative APB 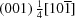, (*d*) non-conservative APB 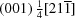 introduced by the rotation of the γ-Al_2_O_3_ unit cell around the 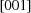 direction, (*e*) non-conservative APB 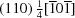 and (*f*) conservative APB 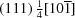. More detailed models are provided in the supporting information.

**Figure 4:**
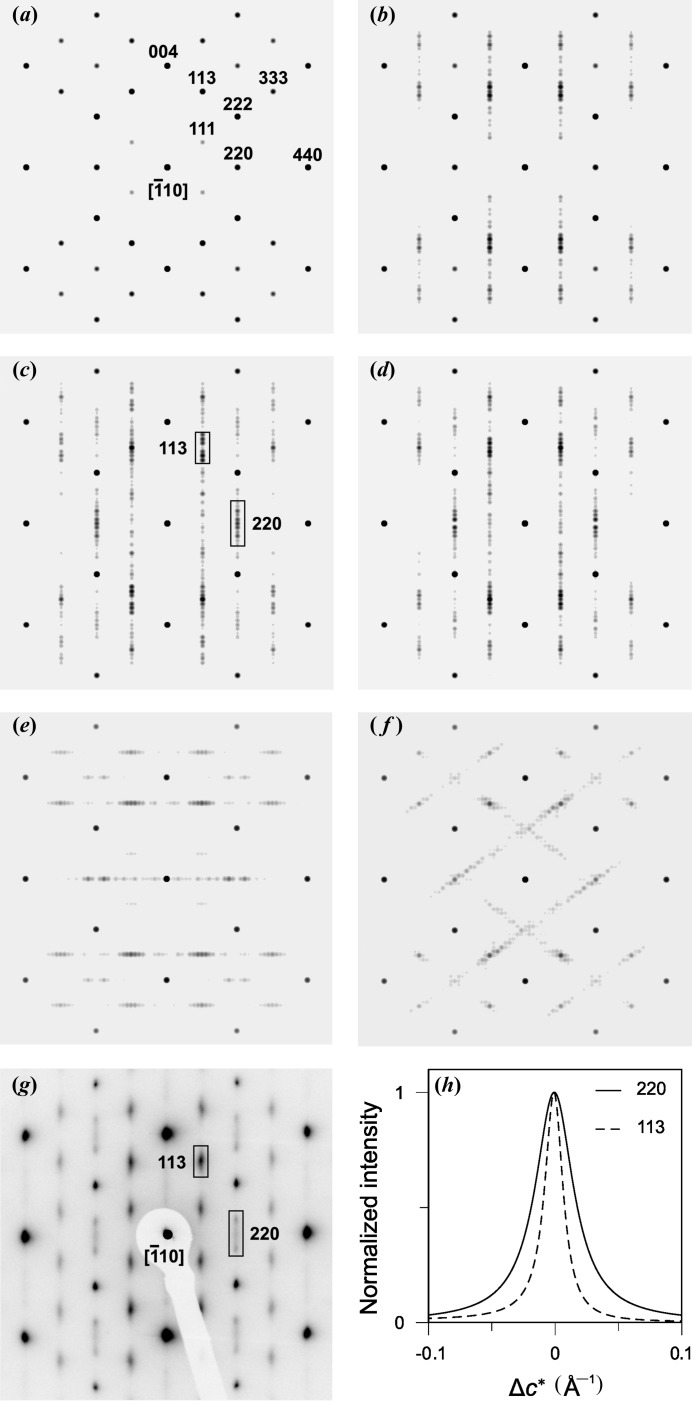
SAED patterns simulated using *JEMS* (Stadelmann, 2012 ▸) for (*a*) a γ-Al_2_O_3_ nanocrystallite with idealized crystal structure, (*b*) with conservative APBs 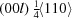, (*c*) with non-conservative APBs 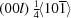, (*d*) with rotational boundaries 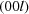 that resemble non-conservative APBs, (*e*) with non-conservative APBs 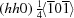 and (*f*) with conservative APBs 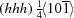 and 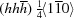. These SAED patterns correspond to the defect types shown in Fig. 3 ▸. (*g*) The experimental SAED pattern once more for comparison. (*h*) The intensity profiles of the reflections 113 and 220 calculated using *DIFFaX* (Treacy *et al.*, 1991 ▸) for non-conservative APBs 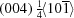 with a probability of 10%.

In our microstructure models derived from non-distorted γ-Al_2_O_3_ (Fig. 3 ▸ *a*), the defect structures were produced by antiphase boundaries (APBs). The examples of the microstructure models include simple glide (Figs. 3 ▸ *b* and 3*f*), which is connected with a shift of the atoms within the glide plane, APBs producing out-of-plane shifts of the atoms (Figs. 3 ▸ *c* and 3*e*) and rotational boundaries (Fig. 3 ▸ *d*). The simple glide, which corresponds to the model suggested by Tsybulya & Kryukova (2008 ▸), produces conservative APBs. The out-of-plane shift, which corresponds to the model proposed by Dauger & Fargeot (1983 ▸), produces non-conservative APBs. Rotational boundaries (RBs) can produce either conservative or non-conservative APBs. The model presented in Fig. 3 ▸(*d*) corresponds to non-conservative APBs. Conservative APBs do not change the local chemical composition, while some of the non-conservative APBs change the local stoichiometry of Al_2_O_3_. The SAED patterns that correspond to the models from Figs. 3 ▸(*a*)–3(*f*) are shown in Figs. 4 ▸(*a*)–4(*f*).

The SAED patterns were simulated using the computer program *JEMS* (Stadelmann, 2012 ▸) for orthorhombic supercells, which contained six APBs of the respective kind. The size of the respective supercell was about 5.6 Å along the electron beam and approximately 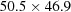
 Å^2^ in the in-plane directions 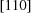 and 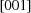, respectively. Thus, the mean distances between the planar defects were approximately 6.7 Å for the APBs from Figs. 3 ▸(*b*)–3(*d*), 7.2 Å for APBs from Fig. 3 ▸(*e*) and 9.9 Å for APBs from Figs. 3 ▸(*e*) and 3(*f*). Such a high defect density suppressed automatically the formation of superstructure reflections.

For simulations of the SAED patterns, the specific APBs from Figs. 3 ▸(*a*)–3(*f*) were complemented by the antiphase boundaries with crystallographically equivalent shifts and gliding or rotation planes. A full set of APBs that belong to the APB system 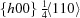 is presented as an example in Table 5. The positions of individual atoms within the supercell were generated as described in Appendix *A* [App appa].

The simulations of the SAED patterns (Figs. 4 ▸ *c* and 4*d*) confirmed that only non-conservative APBs with the gliding planes 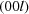 can produce the streaks in the 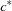 direction and the anisotropic broadening of the diffraction spots, which were observed in the experimental SAED pattern (Fig. 4 ▸ *g*). Conservative APBs 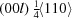 (Fig. 4 ▸ *b*) can be excluded as a possible source of the observed line broadening, as these planar defects do not affect the shape of the reflections with even diffraction indices (220, 224 *etc.*).

The APBs located on the lattice planes 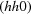, 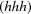 and 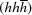 broaden the diffraction spots along the normal direction to the respective lattice plane (Figs. 4 ▸ *e* and 4*f*), thus the presence of these defects can be excluded as well. Analogously, planar defects on other lattice planes can also be eliminated, as they would not broaden the diffraction spots in the 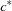 direction.

### 3.2. Possible models of planar defects in γ-Al_2_O_3_ from the point of view of powder XRD   

Although the structural information contained in the SAED pattern of a single crystal is much more comprehensive than the information contained in the powder XRD pattern, the powder XRD patterns were additionally consulted in order to obtain statistically relevant information about the density of microstructure defects. Furthermore, powder XRD provides direct access to all reflections in reciprocal space in contrast to the SAED experiment. Without laborious sample preparation, the plate-like particles were transparent in the electron beam only in the vicinity of the 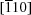 direction, because the facets of the particles have a specific orientation with respect to the crystallographic axes. Consequently, our SAED experiments were limited to the diffraction spots *hhl* (in cubic notation).

**Figure 5:**
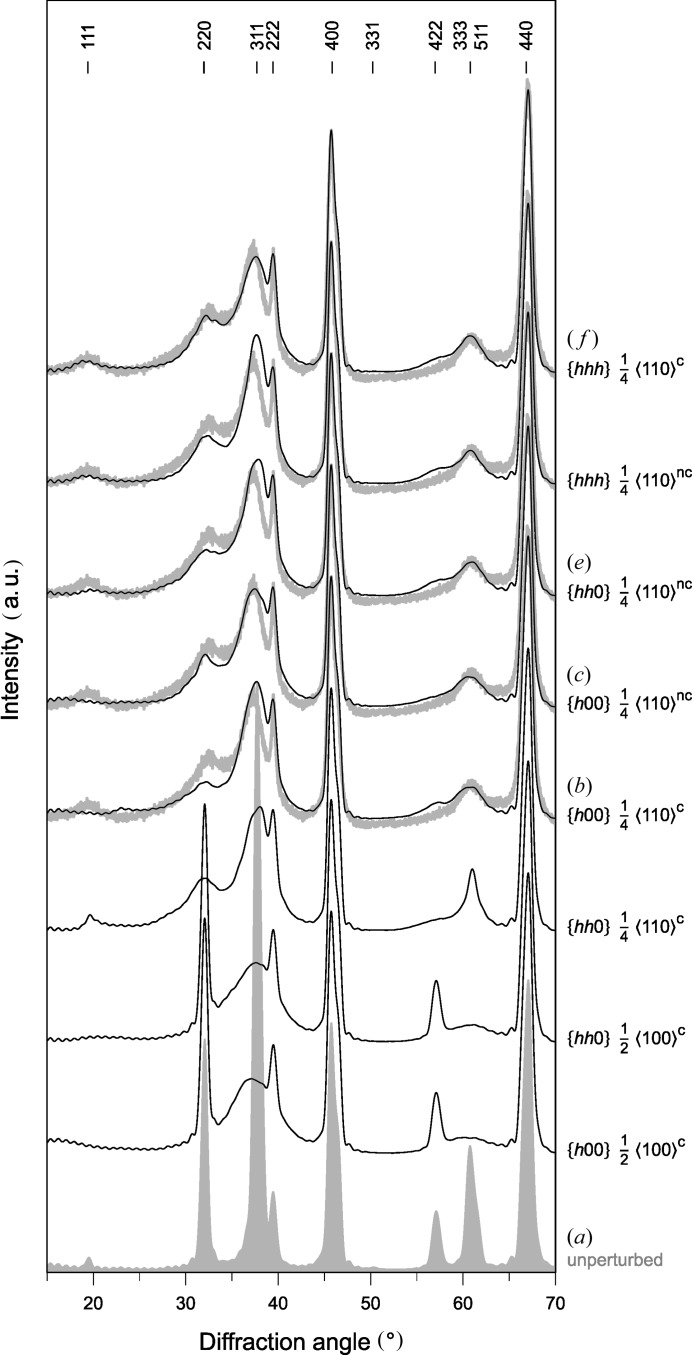
Powder XRD patterns simulated for different APB types (black lines). The patterns, which are labeled (*a*), (*b*), (*c*), (*e*) and (*f*), were simulated using the corresponding models from Fig. 3 ▸. The symbols c and nc denote conservative and non-conservative APBs, respectively. Measured XRD patterns are plotted in gray. For the sake of simplicity, the diffraction lines are labeled with cubic indices. For tetragonal indices, the reader is referred to Table 2 ▸.

The XRD pattern measured in the powder sample of γ-Al_2_O_3_ is displayed in Fig. 5 ▸ together with the XRD patterns simulated for the APBs discussed above. The diffraction patterns in Figs. 5 ▸(*a*), 5(*b*), 5(*c*), 5(*e*) and 5(*f*) were simulated using the same structure models as the SAED patterns in Figs. 4 ▸(*a*), 4(*b*), 4(*c*), 4(*e*) and 4(*f*). Additional XRD patterns were simulated for conservative APBs 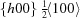, 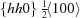 and 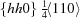 suggested by Tsybulya & Kryukova (2008 ▸), and for non-conservative APBs 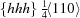. In analogy with the SAED simulations, the powder XRD patterns were simulated by taking into account the crystallographically equivalent APBs. For the APB system 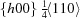, the distribution of the APBs is shown as an example in Fig. 8 and the individual shift vectors are listed in Table 5.

**Figure 6:**
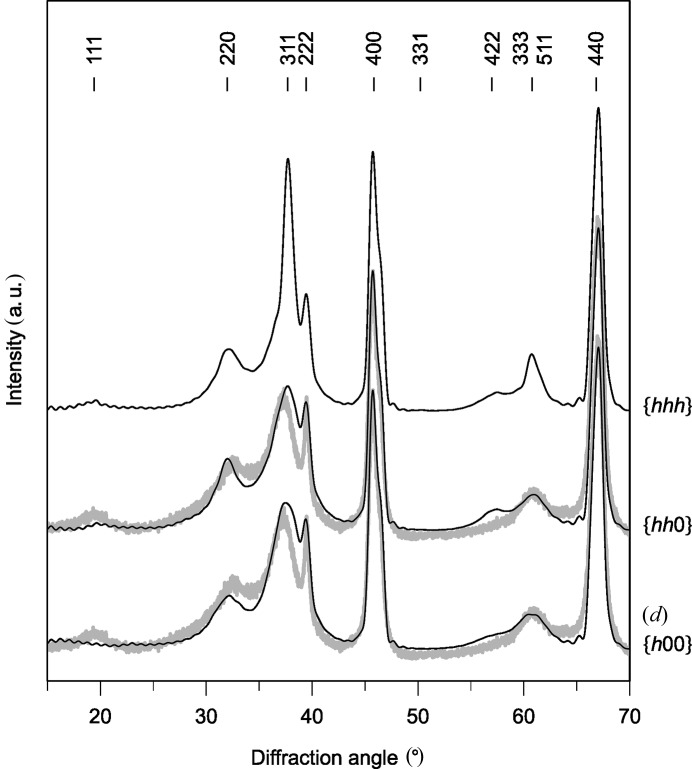
XRD patterns simulated for equally distributed rotational boundaries. The diffraction pattern (*d*) was simulated using the corresponding model from Fig. 3 ▸(*d*).

It follows from the comparison of simulated and measured XRD patterns (Fig. 5 ▸) that conservative APBs 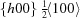, 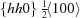 and 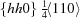 can be excluded from further considerations, because these defects do not reproduce the experimentally observed broadening of diffraction lines 220, 422 and/or 511/333 satisfactorily. On the contrary, the conservative APBs 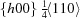 and 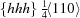 as well as the non-conservative APBs 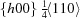, 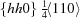 and 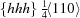 reproduce the observed anisotropic line broadening quite well. The simulated XRD patterns show differences mainly in the intensities of the diffraction lines 111, 311 and 222. The best agreement was achieved for the conservative APBs 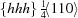. The simulations of the powder diffraction patterns for rotational boundaries (RBs) (Fig. 6 ▸) revealed that RB 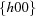 is the most probable defect of this kind.

However, it must be kept in mind that the APBs 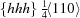, 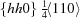 and 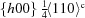 were excluded by SAED simulations, as they broaden the diffraction spots in a wrong direction (Figs. 4 ▸ *e* and 4*f*) or as they do not broaden the diffraction spots 220, 224 *etc.* (Fig. 4 ▸ *b*). Thus, the only promising candidates for description of the planar defects in γ-Al_2_O_3_ derived from boehmite are the non-conservative APBs 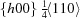 and the RBs 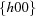.

### 3.3. Quantitative description of planar defects in γ-Al_2_O_3_ based on the analysis of the XRD patterns   

The powder XRD patterns in Fig. 5 ▸ were simulated for cuboidal crystallites with the edge length of 10.8 nm, which contain 24 equally distributed planar defects of the respective kind. The mean distances between the defects were approximately 1.2, 3.1 and 2.7 nm, which corresponds to the planar defect densities of 16.7, 4.5 and 8.3% if the planar defects are located on the planes 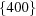, 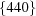 and 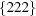, respectively. The size of the cuboidal crystallites (10.8 nm) was determined from the broadening of the ‘narrow’ diffraction lines 222, 400, 440 and 444.

For calculation of the XRD patterns, a fast computer routine (*cuDebye*) was written. It is based on the Debye equation (Debye, 1915 ▸) and runs on a graphic processing unit. Individual atomic positions are generated as described in Appendix *A* [App appa]. The overall isotropic temperature factor [*B* in equation (9)] was ∼2 Å^2^. As an idealized structure was used for the simulations, this factor accounts mainly for static displacements in the structure of γ-Al_2_O_3_. The performance of the simulation routine can be illustrated by the short computing time, which was less than 5 min when calculating the XRD powder pattern with 6500 data points for a structure model consisting of 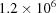 atoms. A drawback of this routine is that it produces intensity oscillations in the low-angle region (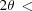 30°) and near the tails of the Bragg peaks (Dopita *et al.*, 2013 ▸).

## 4. Consequences of the proposed structure model of γ-Al_2_O_3_   

### 4.1. Effect of the parameters of the proposed structure model on the XRD patterns   

From the point of view of the kinematical diffraction theory (Warren, 1990 ▸; Guinier, 1994 ▸), one reason for the anisotropic line broadening is a limited coherence of the crystal structure in certain crystallographic directions, which leads to the broadening of the reciprocal lattice points along the corresponding directions in the reciprocal space. In γ-Al_2_O_3_ derived from boehmite, the coherence of the crystal structure is interrupted by planar defects like non-conservative APBs 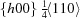 and RBs 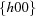, as they modify the atomic ordering and fragment the aluminium sublattice into very small coherent domains. From the crystallographic point of view, the APBs and RBs in γ-Al_2_O_3_ produce disordered polytypes with broad diffraction maxima instead of distinct superstructure satellites. However, as the change in the atomic ordering concerns only the cation (Al^3+^) sites, the diffraction lines stemming from the scattering on oxygen anions are always unaffected.

The change in the atomic ordering is caused by the shift vectors belonging to the APBs. For instance, the non-conservative APBs 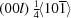 shift some of the Al^3+^ cations from the regular positions 8*a* and 16*d* to 8*b* and 16*c* or even to 48*f*, which corresponds basically to the structure models published by Verwey (1935 ▸), Ushakov & Moroz (1984 ▸), Zhou & Snyder (1991 ▸), Paglia *et al.* (2003 ▸) and Smrčok *et al.* (2006 ▸). However, the crystallographic description of these structure defects implies equal occupancies of crystallographically equivalent atomic positions, while the APBs shift only some of the Al^3+^ cations from their regular positions to the irregular positions. The positions of oxygen anions that occupy the Wyckoff sites 32*e* remain unaffected in all cases.

Frequently, a sequence of APBs 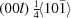 is required to achieve the specific shift vector. The shift of the tetrahedral cations from the Wyckoff sites 8*a* to 8*b* and the shift of the octahedral cations from the Wyckoff sites 16*d* to 16*c* are associated with one of the translation vectors 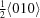, which result, for example, from the sum of the shift vectors 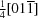 and 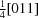. The migration of tetrahedrally coordinated Al^3+^ cations from the Wyckoff sites 8*a* to some of the sites 48*f* is simply produced by a single non-conservative shift of the type 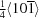, *cf.* Table 6. The shift of the Al^3+^ cations from the 8*a* sites to the remaining 48*f* sites is associated with one of the translation vectors 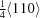 that results, for instance, from the sum of the shift vectors 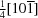 and 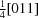.

In comparison with the non-conservative shifts, the conservative shifts of the Al^3+^ cations produce less disorder in the crystal structure. The conservative counterpart of the APBs 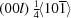 discussed above causes a shift of the Al^3+^ cations from the tetrahedrally coordinated Wyckoff sites 8*a* to 16 of the 48*f* sites only. The 32 atomic positions 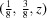, 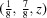, 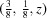, 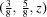, 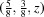, 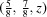, 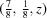 and 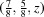, where *z* is an odd multiple of 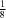, are inaccessible (Fig. 3 ▸ *a*). This example shows that the non-conservative shifts within the APB system 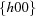 move the Al^3+^ cations to the ‘non-spinel’ positions with a higher probability than the conservative ones.

A consequence of the above atomic shifts is a change in the phase of the scattered wave that primarily modifies the structure factor of a crystallite with APBs and consequently the intensities of diffraction lines. For APBs, the phase shift can be concluded easily from the scalar product of the shift vector of the respective APB with the diffraction vector. The change in the structure factor at the APB is given by the multiplicative factor: 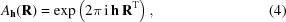
where **h** 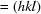 is the vector of diffraction indices and **R** ^T^ is the transposed shift vector. When 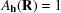, the shift vector does not change the phase of the structure factor at the APB. The domains separated by such APBs are fully coherent and the corresponding diffraction lines are not affected by these defects. The factor 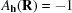 changes the sign of the ‘local’ structure factor, the magnitude of the structure factor calculated over the whole crystallite and finally the diffracted intensity.

An overview of the 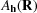 values calculated for heavily broadened diffraction lines 111, 220 and 311 and for possible shift vectors is given in Table 3 ▸. For the narrow diffraction lines 222, 004, 440 and 444, the multiplicative factors calculated for these shift vectors are always equal to unity. It also follows from equation (7) that the structure factors of the diffraction lines 222, 004, 440 and 444 are not affected by the displacement of the aluminium cations from the Wyckoff positions 16*d* to 16*c* and from the Wyckoff positions 8*a* to 8*b* or 48*f*.

As the phase shift and the fragmentation of the cation sublattice are two concurrent consequences of the presence of specific APBs that limit the coherence of the crystal structure from the point of view of the diffraction methods, the probability of the change in the multiplicative factor 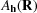 can be related to the broadening of individual diffraction lines. This feature can be illustrated in the different broadening of the reflections 220 and 113 (Figs. 4 ▸ *g* and 4*h*). As can be seen from Table 3 ▸, all crystallographically equivalent shifts producing non-conservative APBs on the lattice planes 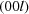 yield negative 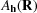 for the reflection 220, while only half of the 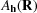 values are negative for the reflections 111 and 113.

At equal probabilities of the crystallographically equivalent shifts, only half of the non-conservative APBs contribute to the broadening of the reflections 111 and 311, while more than half of the crystallographically equivalent shifts ‘broaden’ the reflections of the type 220. The particular reflections 220 and 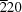 are broadened by all non-conservative shifts of the APBs 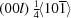, see Table 3 ▸. In other words, the diffraction on the lattice planes 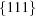 and 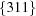 does not recognize half of the non-conservative APBs 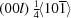 as domain boundaries, while the fraction of these APBs that are active as domain boundaries for the diffraction lines 220 is higher. This phenomenon is apparent in the measured and simulated SAED patterns (Figs. 4 ▸ *c*, 4*g* and 4*h*), where the reflection 220 is approximately two times broader than the reflections 111 and 113.

In powder XRD patterns, the line broadening induced by APBs is additionally modified by the integration of the diffracted intensities over a certain reciprocal-space volume that is usually performed using the powder pattern power theorem (Warren, 1990 ▸). In the case of the APBs 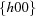, which broaden the reciprocal lattice points along the 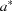, 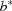 and 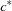 directions, this integration makes strongly broadened diffraction lines asymmetric. Another phenomenon, which additionally affects the broadening and the shape of individual diffraction lines, is the partial coherence of neighboring nano-domains (Rafaja *et al.*, 2004 ▸) that are separated from each other by APBs. Nevertheless, the powder pattern power theorem and the partial coherence of the nano-domains are considered *a priori*, if the Debye equation is utilized for calculation of the diffracted intensities.

The comparison of measured XRD patterns with the XRD patterns simulated for non-conservative APBs 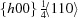 (Fig. 5 ▸ *c*) and RBs 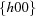 (Fig. 6 ▸ *d*), both having the mean distance of 1.2 nm, shows that the corresponding models can reproduce the defect structure of γ-Al_2_O_3_ derived from boehmite quite well. The only noteworthy discrepancy can be seen for the diffraction line 111, which is extremely weak in the simulated pattern. Partial extinction of this diffraction line is caused mainly by the almost equidistant distribution of APBs and RBs in the microstructure model used for the diffraction pattern simulation. Fragments of the crystal structure separated by a planar defect with a phase change factor 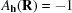 scatter with opposite phases, thus their structure factors (scattered amplitudes) are fully subtracted if these regions have the same size.

An irregular distance between planar defects with negative phase-change factors counteracts this extinction and consequently increases the diffracted intensity. This effect is further enhanced by the partial coherence of neighboring regions, which is the strongest for short diffraction vectors and becomes weaker with increasing magnitude of the diffraction vector (Rafaja *et al.*, 2004 ▸). In this context, it is worth noting that the sequence of the phase-change factors is different for 111 and 311 (Table 3 ▸) and that the magnitude of the diffraction vector is almost twice as large for the diffraction line 311 than for 111, as it follows from 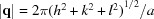. From this point of view, the intensity of the diffraction line 111 is much more sensitive to the regularity of the APB or RB distribution than the intensity of the diffraction line 311.

### 4.2. Influence of the proposed antiphase boundaries on the local stoichiometry of γ-Al_2_O_3_   

From the crystallographic point of view, γ-Al_2_O_3_ crystallizes in a defect spinel structure, in which the Wyckoff positions 32*e* are fully occupied by oxygen and the Wyckoff positions 8*a* and 16*d* partially occupied by aluminium. The partial occupation of the lattice sites is needed to retain the Al_2_O_3_ stoichiometry. On the local scale, where microstructure defects like APBs and RBs are considered, the lattice sites cannot be occupied partially. They can either be fully occupied or stay vacant.

**Figure 7:**
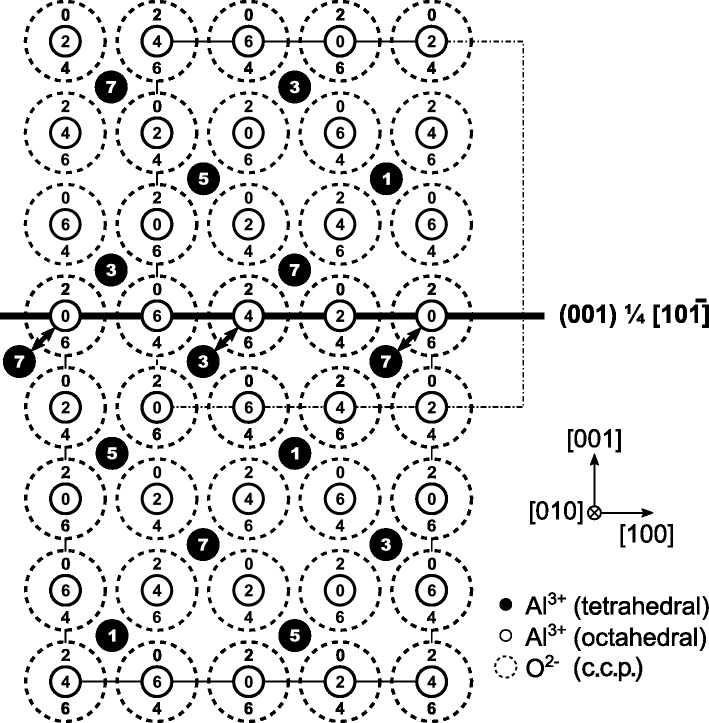
Non-conservative APB 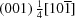 as shown schematically in Fig. 3 ▸(*c*) with the anion and cation positions projected on the plane 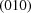. The *y* values of the atomic positions are given in multiples of 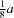. The bidirectional arrows mark the face-sharing tetrahedral and octahedral cations at the APB.

**Figure 8:**
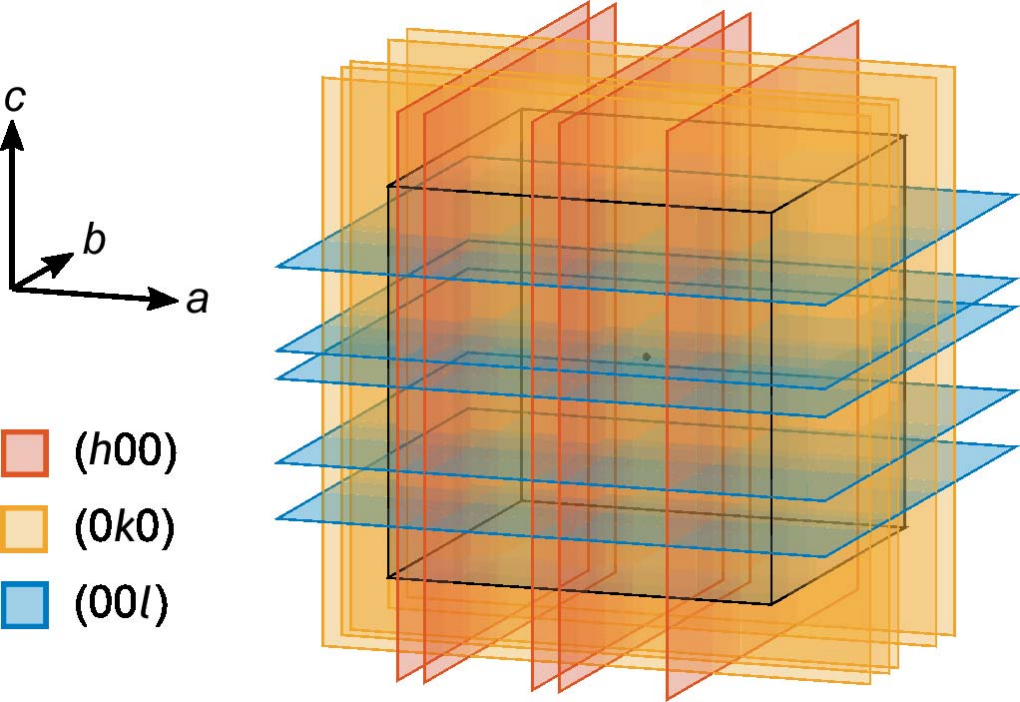
Schematic diagram of planar defects randomly distributed over the lattice planes 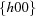 in a γ-Al_2_O_3_ nanocrystallite (black cube).

In the APB model discussed above, the formation of ‘virtual’ vacancies is a necessary consequence of the local lattice shift. As illustrated in Fig. 7 ▸, the non-conservative APB 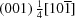 moves the tetrahedral cations located at the Wyckoff positions 8*a* near the antiphase boundary very close to the octahedral cations 16*d* of the original crystal lattice. Thus, the distance between both kinds of the cations would be solely 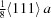, where *a* is the lattice parameter of the cubic structure.

In order to avoid such atomic proximity, one of the two close cations located at the APB must be removed (Cowley, 1953 ▸; Dauger & Fargeot, 1983 ▸; Tsybulya & Kryukova, 2008 ▸). The removal of the cations located within the APBs implies a clustering of vacancies in the vicinity of these defects. Based on the *ab inito* calculations performed by Wolverton & Hass (2000 ▸), the cations at the octahedral sites are preferentially replaced by vacancies in our model. This replacement produces two vacancies per unit area of the APB (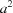). In Fig. 7 ▸, these vacancies are located on a 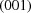 plane. In a general case, two octahedral sites on an 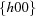 plane remain vacant.

The number of vacancies related to a single APB can be used to estimate the number of APBs (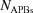) in a single crystallite that are necessary to maintain the stoichiometry of γ-Al_2_O_3_. In the defect spinel structure of γ-alumina, the stoichiometry ratio [Al]/[O] is given by 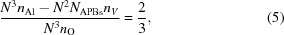
where 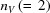 is the number of vacancies per unit area of APB. 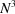 is the number of elementary cells in a cuboidal γ-Al_2_O_3_ crystallite. 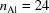 is the number of the Wyckoff positions 8*a* and 16*d* that accommodate Al^3+^ cations and 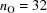 is the number of the Wyckoff positions 32*e* that are occupied by the O^2−^ anions. It follows from equation (5) that the number of APBs scales with the linear size of the crystallite (*N*) as 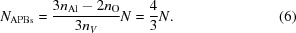
The simulations of the SAED and XRD patterns were performed with cuboidal γ-Al_2_O_3_ crystallites that had the edge length of 10.8 nm and that consisted of 13 to 14 cubic elementary cells with the lattice parameter 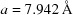. Consequently, the volume of a single crystallite contained about 18 non-conservative APBs 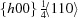.

If the APBs would be equally distributed on all crystallographically equivalent lattice planes 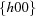, *i.e.* on 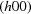, 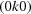 and 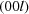, their mean distances would be equal to 1.53 nm. The mean distances of the APBs located only on the lattice planes 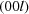 would be 0.56 nm. The theoretical mean distance between the APBs distributed over the crystallographically equivalent lattice planes 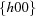, *i.e.* 1.53 nm, is very close to the experimentally determined mean distance of the APBs 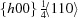 from Section 3.3, which was about 1.2 nm. This comparison indicates that the APBs are located with almost the same probability on all crystallographically equivalent lattice planes 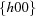.

Finally, it should be noted that the mean distance of APBs is slightly affected by the size of the cuboidal crystallites. As the APBs cannot be located on the crystallite surface in the model their distances become smaller with decreasing crystallite size although their density (number per crystallite volume) is kept constant. For an infinitely large crystallite, the mean distance of APBs 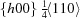 distributed over the lattice planes 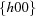 and 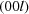 approaches 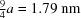 and 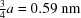, respectively.

### Phase transition γ-Al_2_O_3_ 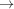 δ-Al_2_O_3_   

4.3.

With increasing temperature and consequently higher cation mobility, the cation vacancies in γ-Al_2_O_3_ start to re­arrange, which leads to the formation of a periodic sequence of APBs that is interpreted as formation of the δ-Al_2_O_3_ supercell (Dauger & Fargeot, 1983 ▸). The periodic ordering of cation vacancies in δ-Al_2_O_3_ leads to the formation of superstructure reflections, which gradually replace the strongly broadened reflections in γ-Al_2_O_3_. The first indications of the superstructure formation are already visible in Fig. 2 ▸. The progress of the continuous transition γ-Al_2_O_3_ 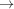 δ-Al_2_O_3_, the formation of several intermediate states between the metastable phases γ-Al_2_O_3_ and δ-Al_2_O_3_, and the completed formation of δ-Al_2_O_3_ at 975^o^C were reported by Rudolph *et al.* (2017 ▸).

An indirect consequence of our model of γ-Al_2_O_3_ is that the atomic ordering in δ-Al_2_O_3_ (as well as the space group and the size of the δ-Al_2_O_3_ unit cell) may depend on the density and distribution of the planar defects in original γ-Al_2_O_3_. As already pointed out by Tsybulya & Kryukova (2008 ▸) and Pakharukova *et al.* (2017 ▸), the distribution of planar defects in γ-Al_2_O_3_ can depend on the synthesis procedure. Therefore, it is not surprising that different unit cells were found for plasma sprayed δ-Al_2_O_3_ and for δ-Al_2_O_3_ derived from boehmite (Levin & Brandon, 1998 ▸).

## 5. Conclusions   

Based on the results of selected-area electron diffraction and powder X-ray diffraction, a defect-structure model of γ-Al_2_O_3_ was proposed. It was shown that in γ-Al_2_O_3_ prepared from boehmite, the dominant defects are non-conservative antiphase boundaries 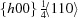 and rotational boundaries 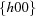, which affect the occupancy of the cation sites in the spinel-like structure of γ-Al_2_O_3_. Both kinds of defect disrupt the coherence of the cation sublattice for diffraction and consequently broaden certain diffraction lines. Furthermore, it was shown that the antiphase boundaries 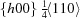 and rotational boundaries 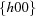 induce a shift in the Al^3+^ cations located in the nano-domains from the Wyckoff sites 8*a* and 16*d* to some of the Wyckoff sites 8*b*, 16*c* and 48*f*, while the positions of the O^2−^ anions remain unaffected.

In contrast to the previously published crystal structure models, in which the Wyckoff sites 8*b*, 16*c* and 48*f* are occupied statistically, the presented model occupies the Wyckoff positions 8*b*, 16*c* and 48*f* non-uniformly.

The shift of atoms caused by the planar defects leads to a convergence of the cations in the vicinity of the planar defects, which is avoided by replacing half of the unfavorably coordinated cations with structural vacancies. The presence of these vacancies retains the stoichiometry of Al_2_O_3_. For quantification of these planar defects, a computer routine based on the Debye scattering equation was written. The comparison of measured and simulated X-ray diffraction patterns revealed that the relevant planar defects in γ-Al_2_O_3_ obtained from boehmite annealed for 20 h at 600^o^C have distances of approximately 1.2 nm and that they are located on all crystallographically equivalent lattice planes 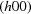, 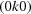 and 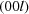.

## Supplementary Material

Information about the supporting structure and video files for Figure 2. DOI: 10.1107/S2052252518015786/ct5007sup1.txt

Video file of Vesta model for Figure 3a. DOI: 10.1107/S2052252518015786/ct5007sup2.mp4

Video file of Vesta model for Figure 3b. DOI: 10.1107/S2052252518015786/ct5007sup3.mp4

Video file of Vesta model for Figure 3c. DOI: 10.1107/S2052252518015786/ct5007sup4.mp4

Video file of Vesta model for Figure 3d-RB. DOI: 10.1107/S2052252518015786/ct5007sup5.mp4

Video file of Vesta model for Figure 3d-APB. DOI: 10.1107/S2052252518015786/ct5007sup6.mp4

Video file of Vesta model for Figure 3e. DOI: 10.1107/S2052252518015786/ct5007sup7.mp4

Video file of Vesta model for Figure 3f. DOI: 10.1107/S2052252518015786/ct5007sup8.mp4

Structure input file for Vesta for Figure 3a. DOI: 10.1107/S2052252518015786/ct5007sup9.txt

Structure input file for Vesta for Figure 3b. DOI: 10.1107/S2052252518015786/ct5007sup10.txt

Structure input file for Vesta for Figure 3c. DOI: 10.1107/S2052252518015786/ct5007sup11.txt

Structure input file for Vesta for Figure 3d-RB. DOI: 10.1107/S2052252518015786/ct5007sup12.txt

Structure input file for Vesta for Figure 3d-APB. DOI: 10.1107/S2052252518015786/ct5007sup13.txt

Structure input file for Vesta for Figure 3e. DOI: 10.1107/S2052252518015786/ct5007sup14.txt

Structure input file for Vesta for Figure 3f. DOI: 10.1107/S2052252518015786/ct5007sup15.txt

## Figures and Tables

**Figure 9:**
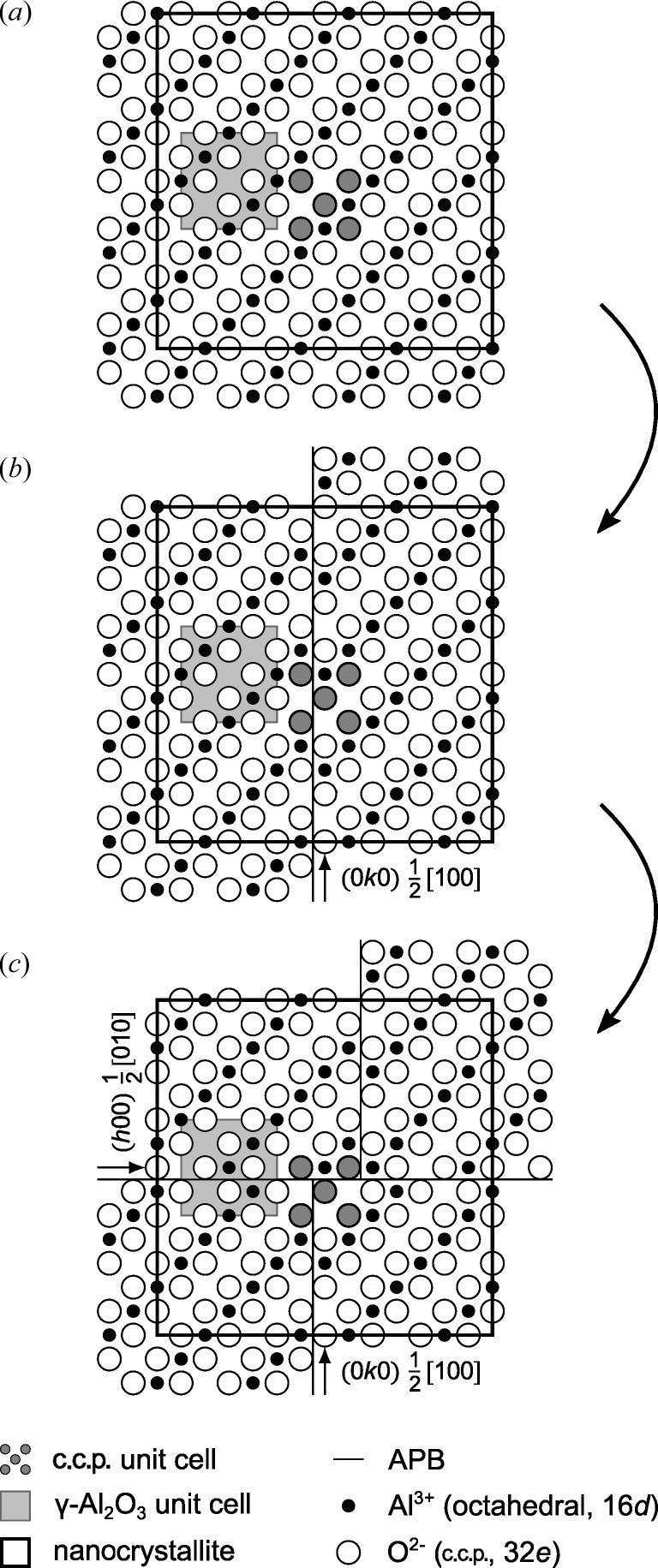
Simplified scheme (a 2D cut in the crystal structure) illustrating the generation of conservative APBs 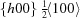. The gray box corresponds to the basal plane of the unit cell of γ-Al_2_O_3_ shown in Fig. 3 ▸(*a*). Empty octahedral positions in the initial unperturbed state (upper panel) correspond to the Wyckoff sites 16*c*.

**Table 1:** Gliding planes in γ-Al_2_O_3_ and the corresponding shift vectors as suggested by Tsybulya & Kryukova (2008 ▸) Resulting stacking faults preserve the atomic ordering within the oxygen sublattice but produce a phase shift within the aluminium sublattice.

Gliding plane	Shift vector
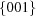	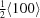 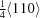
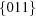	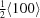 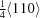
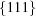	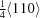

**Table 2:** Equivalence of lattice planes and Wyckoff positions in γ-Al_2_O_3_ that is described either in the space group 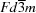 or in the space group 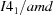 In the tetragonal crystal structure, the interplanar spacings are fully equivalent only if 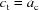. The typical relative difference between 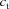 and 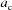 is below 2% (Zhou & Snyder, 1991 ▸).

Lattice planes			Wyckoff positions	
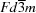		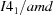	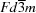	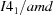
{111}	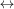	{101}	Aluminium	Tetrahedral position
{220}	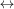	{200}, {112}	8*a*	4*a*
{311}	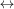	{211}, {103}	8*b*	4*b*
{222}	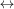	{202}	48*f*	8*e* + 16*g*
{400}	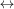	{220}, {004}	Aluminium	Octahedral position
{331}	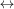	{301}, {213}	16*c*	8*c*
{422}	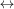	{312}, {204}	16*d*	8*d*
{333}	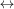	{303}	Oxygen	
{511}	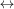	{321}, {105}	32*e*	16*h*
{440}	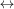	{400}, {224}		

**Table 3:** The phase-change factors 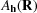 calculated using equation (4) for the crystallographically equivalent shift vectors 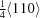 and for the diffraction lines 111, 311 and 220 The phase-change factors are not shown for opposite, *i.e.* ‘negative’, directions, because they yield the same values as the shown directions. The shifts that lead to the non-conservative APBs on the lattice planes 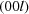 according to Table 6 are highlighted in bold.

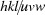	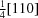	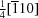	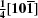	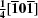	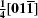	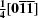
111	−1	1	**1**	**−1 **	**1**	**−1**
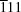	1	−1	**−1**	**1**	**1**	**−1**
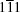	1	−1	**1**	**−1**	**−1**	**1**
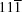	−1	1	**−1**	**1**	**−1**	**1**
113	−1	1	**−1**	**1**	**−1**	**1**
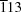	1	−1	**1**	**−1**	**−1**	**1**
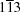	1	−1	**−1**	**1**	**1**	**−1**
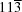	−1	1	**1**	**−1**	**1**	**−1**
131	1	−1	**1**	**−1**	**−1**	**1**
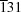	−1	1	**−1**	**1**	**−1**	**1**
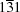	−1	1	**1**	**−1**	**1**	**−1**
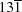	1	−1	**−1**	**1**	**1**	**−1**
311	1	−1	**−1**	**1**	**1**	**−1**
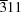	−1	1	**1**	**−1**	**1**	**−1**
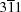	−1	1	**−1**	**1**	**−1**	**1**
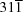	1	−1	**1**	**−1**	**−1**	**1**
220	1	1	**−1**	**−1**	**−1**	**−1**
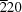	1	1	**−1**	**−1**	**−1**	**−1**
202	−1	−1	**1**	**1**	**−1**	**−1**
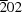	−1	−1	**1**	**1**	**−1**	**−1**
022	−1	−1	**−1**	**−1**	**1**	**1**
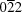	−1	−1	**−1**	**−1**	**1**	**1**

**Table 4:** Positions of Al^3+^ cations and O^2−^ anions in an idealized crystal structure of γ-Al_2_O_3_ based on the cubic space group 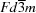 and their occupancies (*O*) Aluminium cations occupy tetrahedral (t) and octahedral (o) positions in a c.c.p. sublattice of oxygen anions.

Element	Wyckoff site	*x*	*y*	*z*	*O*
Aluminium	8*a* (t)	0.125	0.125	0.125	1
	16*d* (o)	0.5	0.5	0.5	0.833
	8*b* (t)	0.375	0.375	0.375	—
	16*c* (o)	0	0	0	—
	48*f* (t)	0.375	0.125	0.125	—
					
Oxygen	32*e*	0.25	0.25	0.25	1

**Table 5:** List of the APB types and individual APBs for the APB systems 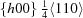 and 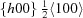 Conservative and non-conservative APBs are denoted by c and nc, respectively. Opposite directions are not shown.

APB system	APB types on the equivalent planes	Distinct APBs
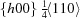	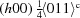	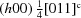
		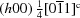
	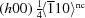	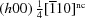
		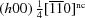
		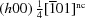
		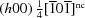
	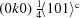	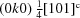
		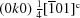
	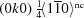	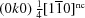
		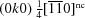
		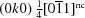
		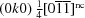
	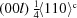	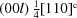
		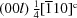
	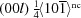	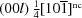
		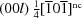
		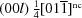
		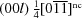
		
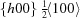	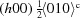	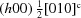
		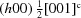
	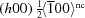	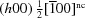
	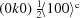	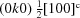
		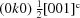
	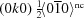	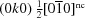
	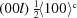	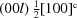
		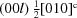
	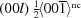	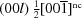

**Table 6:** Possible conservative (c) and non-conservative (nc) APBs located on the lattice planes 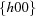, 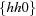 and 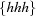 and the corresponding shift vectors 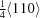 Opposite directions are not given, as they lead to the same APB types.

Plane/shift	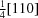	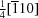	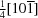	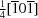	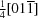	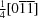
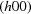	nc	nc	nc	nc	c	c
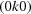	nc	nc	c	c	nc	nc
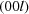	c	c	nc	nc	nc	nc
						
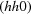	nc	c	nc	nc	nc	nc
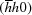	c	nc	nc	nc	nc	nc
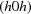	nc	nc	c	nc	nc	nc
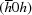	nc	nc	nc	c	nc	nc
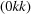	nc	nc	nc	nc	c	nc
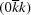	nc	nc	nc	nc	nc	c
						
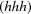	nc	c	c	nc	c	nc
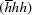	c	nc	nc	c	c	nc
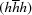	c	nc	c	nc	nc	c
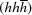	nc	c	nc	c	nc	c

## Appendix A Modeling of planar defects and simulation of X-ray diffraction patterns

The presented structure model of γ-Al_2_O_3_ is based on an idealized cubic spinel structure with the space group , in which the Wyckoff positions 8*a* and 16*d* are occupied by aluminium cations and the Wyckoff positions 32*e* by oxygen anions. The fractional coordinates of the idealized structure are given in Table 4 ▸. The partial occupancy of the Wyckoff position 16*d* balances the stoichiometry of Al_2_O_3_.

The Wyckoff positions 8*b*, 16*c* and 48*f* can be found with a marginal occupancy in several structure reports (Verwey, 1935 ▸; Ushakov & Moroz, 1984 ▸; Zhou & Snyder, 1991 ▸; Paglia *et al.*, 2003 ▸; Smrčok *et al.*, 2006 ▸). Partial occupancy of these Wyckoff positions modifies the structure factors (and intensities) of the diffraction lines with odd indices, and the structure factors and intensities of the diffraction lines 220, 422, 442 *etc.* see the following equation:

In this equation,  and  are the atomic scattering factors of aluminium and oxygen, and *O* is the occupancy of the corresponding Wyckoff site. For the modeling of the planar defects, a *Matlab* routine was written. In this routine, the planar defects are generated on the respective lattice planes *i.e.* ,  or . The planar defects are distributed randomly with the desired probability over the respective lattice planes, as it is illustrated in Fig. 7 ▸ for lattice planes . In the next step, the respective shift vectors were applied, as shown in the example in Fig. 8 ▸ for conservative antiphase boundaries . In the case of non-conservative shifts, the overlapping parts of the structure are considered only once.

The APBs  from Fig. 8 ▸ move the aluminium cations from the Wyckoff positions 16*d* (small filled circles) to the Wyckoff positions 16*c* (empty positions between the oxygen anions). The Wyckoff positions 32*e* occupied by oxygen anions (large open circles) are unaffected by all planar defects under consideration.

The APBs  were chosen, because they can be depicted quite demonstratively in 2D. The entire set of the shift vectors belonging to the respective lattice plane is shown in Table 5 ▸. The kind of APB (conservative or non-conservative) is distinguished by the respective upper index. The APBs located on other lattice planes are classified in Table 6 ▸.

Successive application of such sublattice translations tessellates the original γ-Al_2_O_3_ crystallites into small domains (thin lines in Fig. 9 ▸), which can scatter both in the same phase or in the antiphase, as discussed above and illustrated in Table 3 ▸. When the rotational boundaries are modeled, the lattice planes defining the positions of these planar defects are set up in a similar way to the antiphase boundaries. However, the respective rotation axis, *i.e.* two-, three- and fourfold for the RBs ,  and , is applied instead of the shift vector.

The atomic positions within the nanocrystallites were simulated for the crystallite size of 10.8 nm, which was determined from the width of the ‘narrow’ diffraction lines 222, 400, 440 and 444. This size corresponds to the size of the coherent oxygen sublattice. For the typical defect densities, such a crystallite contains between 100 and 1000 nano-domains of different shape and size. The planar defects were modeled for a cubic structure with the elementary cell size of *a* = 7.942 Å, which was afterwards tetragonally distorted in order to arrive at the measured *c*/*a* ratio of 0.985.

The XRD patterns were calculated with the aid of the general scattering equation (Debye, 1915 ▸)

which intrinsically accounts for the powder pattern power theorem and for the partial coherence of nano-domains in a nanocrystallite. In equation (8), *q* is the magnitude of the diffraction vector (),  is the diffraction angle and λ is the wavelength of the X-rays.  denotes the distances between the atoms *i* and *j* within the crystallite. The factor  contains the atomic scattering factors *f*, the occupancies *O* and the temperature factors *B* for each atomic pair

The temperature factors were assumed to be isotropic and equal for all atoms. For comparison with the measured XRD patterns, the intensities calculated using equation (8) were corrected for polarization. Other effects including instrumental broadening were neglected (Dopita *et al.*, 2015 ▸).

In order to account for a random distribution of the planar defects in powder samples, in which several crystallites can diffract concurrently, the planar defects in a crystallite were generated ten times at random positions but with the same type of defect and with the same defect density, and summed. The resulting simulated intensities were averaged, before they were compared with the experimental data.

The intensity calculation was speeded up by dividing the atoms in equation (8) into subgroups with the same properties () and distances ():

 means the frequency of the distance . Moreover, as the oxygen sublattice remains unchanged in the faulted structure of γ-Al_2_O_3_, only the contribution of the aluminium sublattice to the diffracted intensities has to be recalculated for each kind and density of microstructure defect, when this procedure is utilized.

Consequently, the most time-consuming step is the calculation of the distribution of the atomic distances. However, when running on a CUDA capable graphic processing unit (GTX 980 TI from NVIDIA), our C++/Cuda routine *cuDebye* needs less than 5 min to simulate the X-ray powder diffraction pattern from nanocrystallites containing approximately  atoms. The experimental code of *cuDebye* that was written in C++ using *Visual Studio Community* 2015 and the CUDA Toolkit 8.0 is deposited at GitHub (https://github.com/Martin-Rudolph/cuDebye).
